# Trace metals and over-expression of metallothioneins in bladder tumoral lesions: a case-control study

**DOI:** 10.1186/1746-6148-5-24

**Published:** 2009-07-18

**Authors:** André FS Amaral, Teresa Cymbron, Fátima Gärtner, Manuela Lima, Armindo S Rodrigues

**Affiliations:** 1 Spanish National Cancer Research Centre (CNIO), Genetic and Molecular Epidemiology Group, Madrid, Spain; 2 CIRN, DB, University of the Azores, APT 1422, 9501-855 Ponta Delgada, Portugal; 3 PHERG, University of the Azores, APT 1422, 9501-855 Ponta Delgada, Portugal; 4 IBMC-Institute for Molecular and Cell Biology, University of Porto, 4150-180 Porto, Portugal; 5 IPATIMUP, R. Dr. Roberto Frias, s/n, University of Porto, 4200-456 Porto, Portugal

## Abstract

**Background:**

Previous studies have provided some evidence of a possible association between cancer and metallothioneins. Whether this relates to an exposure to carcinogenic metals remains unclear.

**Methods:**

In order to examine the association between the expression of metallothioneins and bladder tumors, and to compare the levels of arsenic, cadmium, chromium, lead and nickel in animals with bladder tumors and animals without bladder tumors, 37 cases of bovine bladder tumors and 17 controls were collected. The detection and quantification of metallothioneins in bladder tissue of both cases and controls was performed by immunohistochemistry. And the quantification of metals in tissue and hair was assessed by inductively coupled plasma – mass spectrometry.

**Results:**

Increased expression of metallothioneins was associated with bladder tumors when compared with non-tumoral bladder tissue (OR = 9.3, 95% CI: 1.0 – 480). The concentrations of cadmium, chromium, lead and nickel in hair of cases were significantly higher than those of controls. However, as for the concentration of metals in bladder tissue, the differences were not significant.

**Conclusion:**

Though the sample size was small, the present study shows an association between bladder tumors and metallothioneins. Moreover, it shows that concentrations of metals such as cadmium, chromium, lead and nickel in hair may be used as a biomarker of exposure.

## Background

In S. Miguel Island (Azores, Portugal), around 10% of the adult cattle slaughtered do not enter the human food-chain on the basis of suspected urinary bladder tumors [1]. The occurrence of urinary bladder cancer in cattle has been related to infection by bovine papilloma virus (BPV), and grazing on pastures rich in bracken fern [2]. Though these have been mentioned as potential risk factors of urinary bladder cancer in cattle, other factors may be of importance. In humans, and besides tobacco, other environmental factors play a role in the development of bladder cancer. These are aromatic amines, polycyclic aromatic hydrocarbons, chlorination by-products, and high arsenic levels[3].

Other trace metals besides arsenic, such as lead and cadmium, are known carcinogens that have already been associated to certain types of cancer [4]. They mainly act by inducing oxidative stress, which activates several proto-oncogenes, like FOS and MYC, and enhances the DNA binding activity of several transcription factors, such as NF-κB and AP-1 [5,6]. DNA damage in the form of strand breaks and adducts can also arise as result of metal-induced stress [7,8]. Arsenic per se has been shown to induce tumors in mice without prior or later exposure to other chemical or physical agents [9], it acts more in the cytoplasm than in the nucleus, and it only causes DNA damage when present at high concentrations. Cadmium is involved in gene mutation, stimulation of cell proliferation, and progression of tumor cells. Chronic exposure to these and other metals, resulting in continued bioaccumulation, has been linked not only to mutagenic and carcinogenic effects but also to the induction of metallothioneins, in several tissues and organs [10-12]. Chromium and nickel also can induce oxidative stress, DNA damage and increased activity of transcription factors [13-15]. Lead may be more a promoter rather than an initiator of cancer, since it reduces the ability of the cell to protect or repair DNA damaged by other exposures [16].

The use of biomarkers such as metal levels in hair, nails, blood or urine is one of the ways of detecting abnormal levels of these elements in the organism. And while blood and urine give an idea of recent exposure, hair and nails are proxies of longer time-frame exposures [17-19]. Another way, in particular, for quantifying the levels of metals in solid tissues that one may use is the immunohistochemistry of metallothioneins. Metallothioneins (MTs) are low-molecular weight (6–7 kDa) and cysteine-rich proteins that normally bind group 1B and 2B metals. These proteins are mainly involved in the homeostasis and storage of copper and zinc, but also play a protective role against the toxic effects of other metals and act as stress-response proteins [20-22]. MTs have been previously suggested to be also associated to several types of cancer, and MT-positive cells seem to have a higher activity of proliferation leading to more aggressiveness of the tumor and decreased survival rates [23-25].

The aims of this pilot study were: (i) to examine the association between the expression of metallothioneins and bladder tumors; and (ii) to compare the levels of arsenic, cadmium, chromium, lead and nickel in animals with bladder tumors and animals without bladder tumors.

## Methods

### Animals, Slaughterhouse Procedures and Samples

Systematic opening and mucosal macroscopic inspection of urinary bladder of cattle killed in the main slaughterhouse in S. Miguel Island (Azores, Portugal) is carried out since 1991. As part of a larger project aiming to establish a genomic and histological bank of *Bos taurus*, comprising healthy animals and animals with tumors, from February 2007 to October 2007, 54 urinary bladders of female adult grazing Holstein Friesian cattle slaughtered there were sampled. The animals were 8 ± 0.3 years-old, and were coming from all over S. Miguel Island. After a preliminary macroscopic analysis meant to establish the groups of bladder lesions and controls, urinary bladders were sent to the laboratory. From each urinary bladder two adjacent tissue samples were taken, and while one was fixed in 10% neutral buffered formalin and paraffin embedded for histopathology and immunohistochemistry, the other was prepared for inductively coupled plasma – mass spectrometry (ICP/MS) analysis, in order to quantify the concentrations of arsenic, cadmium and lead. From 36 animals, samples of hair were available and were used for ICP/MS quantification of abovementioned metals.

### Histopathology

Paraffin embedded tissues were sectioned (4 μm) and stained with haematoxylin and eosin. Samples were histologically classified by a pathologist, and tumoral cases were discriminated from controls according to the World Health Organization international histological classification of urinary bladder tumors of domestic animals [26].

### MT immunohistochemistry

Sections were dewaxed in xylene, hydrated in acetone, brought to distilled water and washed in PBS. Endogenous peroxidase activity was quenched by shortly incubating the sections in 3% hydrogen peroxide. Sections were then washed in PBS and incubated at room temperature, inside a moist chamber, for 30 min with a blocking solution, which was made of 5% normal goat serum diluted in PBS. After a brief rinse in PBS, sections were incubated overnight, inside a moist chamber, at 4°C with the antibody Mouse Anti-Metallothionein, clone E9, (Zymed) diluted (1:100) in PBS. After several rinses in PBS, sections were incubated during 1 h at 4°C, inside a moist chamber, with the antibody Goat Anti-Mouse IgG (Sigma) diluted (1:20) in PBS. Then rinsed in PBS, incubated with ExtrAvidinTM (Sigma) (1:20) in PBS, for 30 min. Following several rinses in PBS, the visualization of peroxidase activity was achieved using 3,3'-diaminobenzidine tetrahydrochloride (DAB) (Sigma). Finally, after a brief rinse in PBS, sections were counterstained with haematoxylin (5–10 s), dehydrated in ethanol, cleared in xylene and mounted in DPX. In control sections, PBS was used instead of the primary antibody solution. The semi-quantitative assessment of the MT immunohistochemical levels found on tissue sections was performed on a consensus basis by two observers (A.A. and A.R.) using a Sony CCD-Iris camera coupled to a Laborlux S (Leitz) light microscope. After establishing the criteria to state the consensus basis, a previous trial was done with no significant differences among the results obtained by the two observers. According to a method previously described [25,27], the percentage of stained cells (staining score) was graded as follows: 0 (no staining); 1 (>0 to 5%); 2 (>5 to 50%); 3 (>50%). Additionally, an intensity-distribution index (MT.idi) was calculated by multiplying, for each case, the staining score by the staining intensity (weak = 1; moderate = 2; strong = 3).

### Metal analysis

The bladder tissue samples were dried (130°C) for 48 hours, digested in aqua regia 86 at 95°C for 2 hours and microwave digested inside closed vessels. Hair samples had the same treatment as bladder tissue samples, but were previously brushed and washed in a sequence of acetone, bi-distilled water, and acetone [28] to remove external deposition of feces and dust. Resultant sample solutions were diluted and analyzed on a Finnegan Mat Element 2 High Resolution ICP/MS (Actlabs, Canada) for the quantification of arsenic, cadmium, chromium, lead, and nickel. For quality control, internal standards and reference materials were run together with the samples, and not less than six different reference materials covering all the elements in study were used. Duplicate samples were used as well in order to determine precision of the analysis. For each element, a minimum of three standards were used to cover the analytical working range of the instrument. Ultrapure water was used to prepare blanks and calibration standards, and three replicate assessments were performed for each sample.

### Statistical analysis

Differences in metal concentrations in hair and bladder samples between lesions and controls cases were examined using the Mann-Whitney U test and considered significant when p ≤ 0.05. To examine the association between over-expression of MTs and bladder tumors, odds ratio (OR) and Fisher's exact test was used and considered significant also when p ≤ 0.05. Statistical analyses were performed with R 2.7.0 [29].

## Results

After histological confirmation, two groups of animals were obtained, one of 37 cases, comprising only non-muscle invasive tumors, and one of 17 controls, without tumors. The former included 18 carcinomas, 8 papillomas, 6 adenomas, and 5 hemangiomas.

### MT over-expression

**Figure 1:**
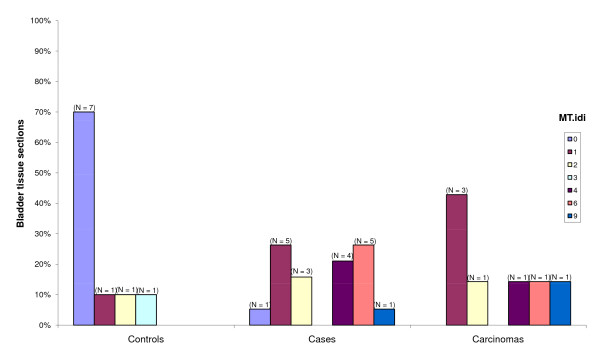
**MT expression in bladder tissue from controls and case lesions**. Controls show higher percentage of bladder tissue sections negatively stained for metallothioneins than cases. MT.idi values range from zero (no staining) to 9 (>50% of cells, strongly stained).

Over-expression of MT in the bladder tissue was defined has a value of MT.idi above two. According to this, it was observed that the probability of over-expression of MT in bladder tumor tissue was 9.3 times significantly higher than in normal bladder tissue, which indicates an association between the levels of expression of MT and bladder tumors, regardless of the type of tumor. In carcinomas, the probability of increased expression of MT was 5.96 times higher than in non-tumoral tissue (p = 0.25) (Figure 1; Table 1).

**Table 1:** MT over-expression in bladder tissue from controls and case lesions.

	MT.idi ≤ 2	MT.idi > 2	OR (95% CI)	p-value
Controls (n = 10)	90%	10%	Reference	-
All cases (n = 19)	47.3%	52.7%	9.3 (1.0 – 480)	0.043
Carcinomas (n = 7)	57.1%	42.9%	5.96 (0.35–392)	0.25

Magnitude of association between MT over-expression and cases is expressed as odds ratio (OR), and its 95% confidence interval (95% CI).

### Metal analysis

In general and when comparing hair samples, the concentrations of all five metals were higher in cases than in controls. Moreover and apart from arsenic, the concentrations of the cadmium, chromium, lead and nickel in case lesions were significantly higher than in controls (Table 2). In fact, the concentrations of chromium, lead and nickel in cases were approximately five-, two-, and three-fold higher than those from controls. The concentrations of chromium and nickel were also significantly higher in those with carcinomas when compared to controls (Table 2).

**Table 2:** Concentrations (mean μg.kg^-1 ^± standard error) of arsenic, cadmium, chromium, lead and nickel as measured in hair and bladder tissue, from controls and case lesions.

	Arsenic	Cadmium	Chromium	Lead	Nickel
Bladder tissue					
Controls (n = 17)	19.94 ± 6.46	21.60 ± 2.02	269.41 ± 38.18	10.29 ± 1.84	0.08 ± 0.03
All cases (n = 37)	23.00 ± 2.62	25.91 ± 1.95	247.84 ± 33.36	14.73 ± 2.87	0.07 ± 0.01
Carcinomas (n = 18)	17.94 ± 4.21	20.83 ± 2.13	283.89 ± 41.85	13.33 ± 3.77	0.06 ± 0.01

Hair					
Controls (n = 13)	59.54 ± 12.44	16.25 ± 3.01	123.85 ± 25.83	460.77 ± 176.78	0.09 ± 0.02
All cases (n = 23)	61.96 ± 8.25	23.20 ± 2.39^a^	634.35 ± 127.78^a^	1095.22 ± 214.77^a^	0.30 ± 0.05^a^
Carcinomas (n = 10)	58.30 ± 12.69	21.61 ± 3.44	559.00 ± 198.40^a^	960.00 ± 318.04	0.28 ± 0.08^a^

^a ^differs significantly (p ≤ 0.05) from controls.

As measured in bladder tissue, concentrations of arsenic, cadmium, chromium, lead and nickel in case lesions were not significantly different than in controls (Table 2).

## Discussion

The entire list of risk factors determining the development of bladder cancer, either in humans or in cattle, remains unclear and little is known about the role of carcinogenic metals in this type of cancer. Also, the relation between MT over-expression and tumors is not fully understood yet.

In the present study, a high association between over-expression of MT in the bladder tissue and tumors was observed. Other studies, also with small sample size, have already shown some association between these two variables, both *in vitro *and *in vivo *[30-32]. Although the expression of MT has been reported to occur under stress imposed by organic compounds [31], MT are mostly known for being related to the metabolism of toxic metals [20].

Regarding case lesions, only arsenic, cadmium and nickel were slightly higher in tumors than in non-tumors. The higher concentrations of these carcinogenic metals in the bladder tissue of cases could to some point explain the over-expression of MT, especially in the case of cadmium which is known to induce the expression of MT [33,34]. However, the concentrations of these metals were not significantly different between cases and controls, what may lead one to suggest that the levels of these elements in the bladder tissue at the moment of diagnosis are probably not representative of past exposure. But, this may even though be the reason for the high expression levels of MT, since the synthesis of this protein seems to be maintained in the tissue for long periods after the initial exposure, acting both as detoxifying and preventive mechanisms [34,35].

As measured in hair, the concentrations of all the metals considered, with the exception of arsenic, in cases were significantly higher than in controls. Thus, with hair samples it was possible to distinguish between cases and controls, as opposite to what was observed with bladder tissue. Another striking aspect of the distribution of the metals in cases is the fact that the concentrations of arsenic, chromium, lead, and nickel in hair were approximately 3-, 3-, more than 70-, and 3-fold higher than in bladder tissue, respectively. Internally, the liver and the kidney are the main target organs for accumulation of toxic metals, independently of their carcinogenic properties. Therefore, the bladder does not usually presents high concentrations of metals. On the other hand, hair is one of the matrixes through which the organism excretes excess of metals, either essential or non-essential. Hair is in fact considered a good matrix to be used as a biomarker of exposure to metals, and a good proxy of long term exposures [36,37]. The concentrations of cadmium and lead in hair of cattle with bladder tumors from the present study were below those of normal cattle reared around different industrial/urban areas [38], and yet the concentrations of lead and chromium were above the levels of those from unpolluted areas [39,40]. Overall, the concentrations of metals found in cases render more investigation on the mechanisms of carcinogenic metals and their role in the development of bladder cancer.

Though the prevalence of BPV was not assessed in the present study, there is previous evidence showing that its prevalence in the bladder mucosa, from cattle of S. Miguel Island, with and without tumoral lesions is 42% and 43%, respectively, which suggests that this factor is probably not acting as a confounder [41]. Also, the fact that these animals are randomly coming from several herds from S. Miguel Island should imply the lack of a differential exposure to bracken fern.

## Conclusion

Despite the small sample size of the present study, an association between MT over-expression and bladder tumors was shown. However, more studies with larger and more homogeneous samples are needed to verify this relation, and to examine potential associations between arsenic, cadmium, chromium, lead, and nickel that were seen to be in higher concentrations in tumors. Larger samples will also allow for stratification by sub-types of bladder cancer. Also, the present study highlights the potential of hair as a matrix to assess exposure to carcinogenic metals, and concentrations of metals in biological samples as useful biomarkers of exposure and disease development in cattle.

## Competing interests

The authors declare that they have no competing interests.

## Authors' contributions

AFSA participated in the design of the study, carried out the immunohistochemical procedures, performed the statistical analysis and drafted the manuscript. TC carried the sampling and histological preparation. FG carried out the histological characterization of the tumor types. ML coordinated the pilot study and helped to draft the manuscript. ASR participated in the design and coordination of the study, and helped to draft the manuscript. All authors read and approved the final manuscript.
