# Trace metals and over-expression of metallothioneins in bladder tumoral lesions: a case-control study

**DOI:** 10.1186/1746-6148-5-40

**Published:** 2009-11-11

**Authors:** André FS Amaral, Teresa Cymbron, Fátima Gärtner, Manuela Lima, Armindo S Rodrigues

**Affiliations:** 1 Spanish National Cancer Research Centre (CNIO), Genetic and Molecular Epidemiology Group, Madrid, Spain; 2 CIRN, DB, University of the Azores, APT 1422, 9501-855 Ponta Delgada, Portugal; 3 PHERG, University of the Azores, APT 1422, 9501-855 Ponta Delgada, Portugal; 4 IBMC-Institute for Molecular and Cell Biology, University of Porto, 4150-180 Porto, Portugal; 5 IPATIMUP, R. Dr. Roberto Frias, s/n, University of Porto, 4200-456 Porto, Portugal

## Correction

During the publication process of this work [1], we noted that the column for nickel in table 2 was not showing the concentration values in the proper units. This change has been made in the updated table (see table 2).

**Table 2:** Concentrations (mean μg.kg^-1 ^± standard error) of arsenic, cadmium, chromium, lead and nickel as measured in hair and bladder tissue, from controls and case lesions.

	**Arsenic**	**Cadmium**	**Chromium**	**Lead**	**Nickel**
Bladder tissue					
Controls (n = 17)	19.94 ± 6.46	21.60 ± 2.02	269.41 ± 38.18	10.29 ± 1.84	80 ± 30
All cases (n = 37)	23.00 ± 2.62	25.91 ± 1.95	247.84 ± 33.36	14.73 ± 2.87	70 ± 10
Carcinomas (n = 18)	17.94 ± 4.21	20.83 ± 2.13	283.89 ± 41.85	13.33 ± 3.77	60 ± 10

Hair					
Controls (n = 13)	59.54 ± 12.44	16.25 ± 3.01	123.85 ± 25.83	460.77 ± 176.78	90 ± 20
All cases (n = 23)	61.96 ± 8.25	23.20 ± 2.39^a^	634.35 ± 127.78^a^	1095.22 ± 214.77^a^	300 ± 50^a^
Carcinomas (n = 10)	58.30 ± 12.69	21.61 ± 3.44	559.00 ± 198.40^a^	960.00 ± 318.04	280 ± 80^a^

^a ^differs significantly (p ≤ 0.05) from controls.
